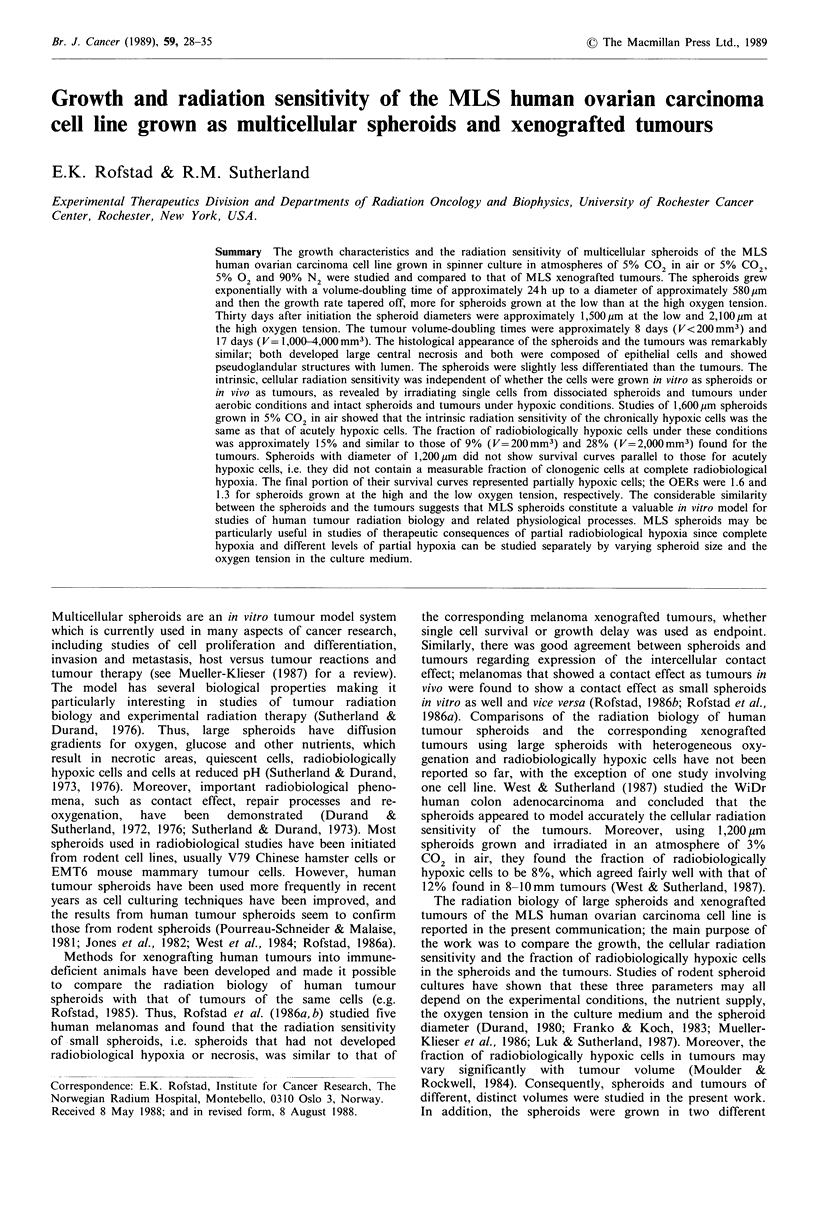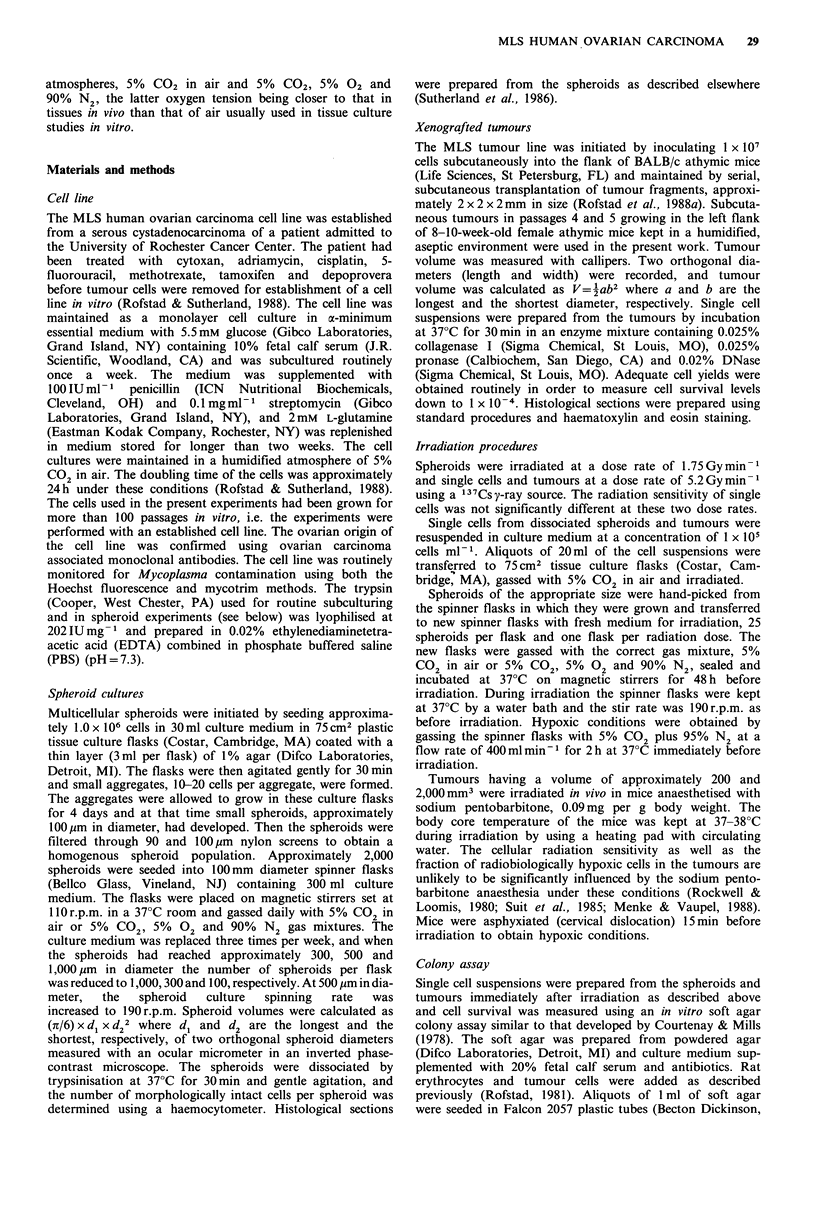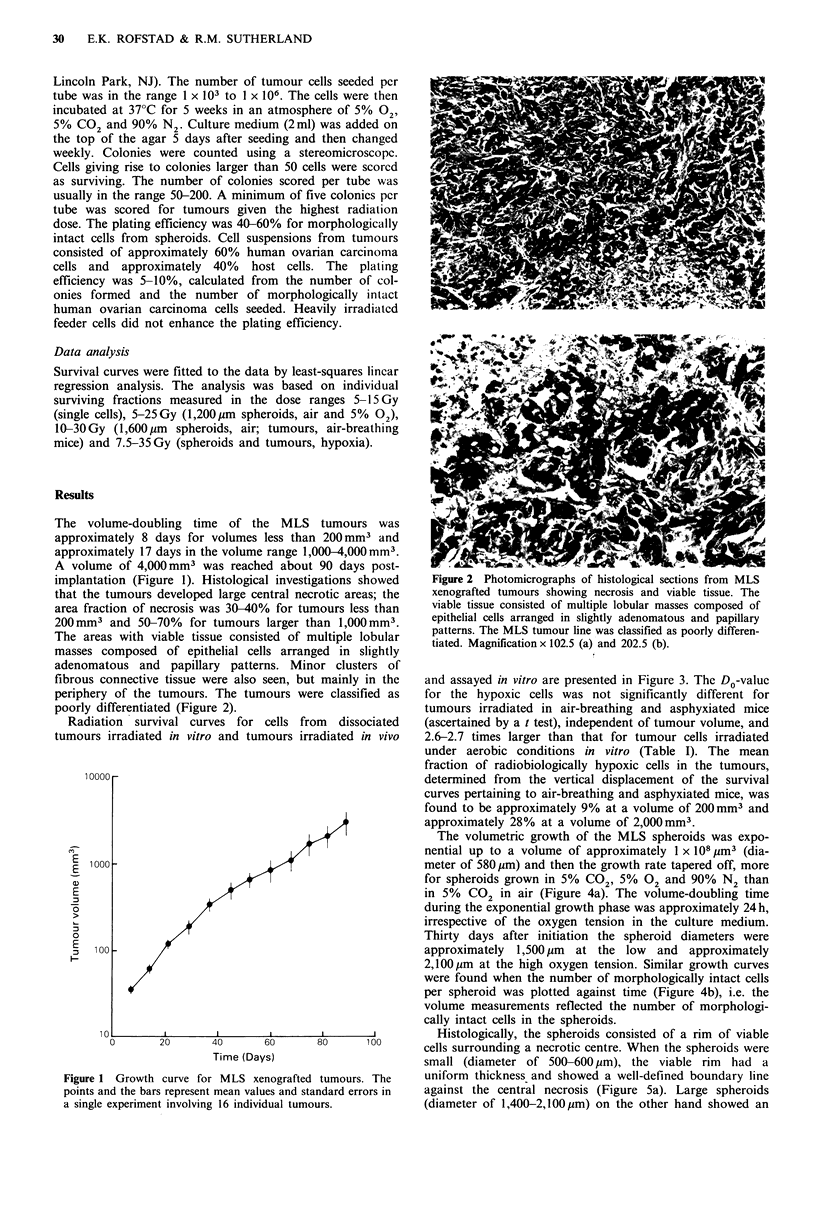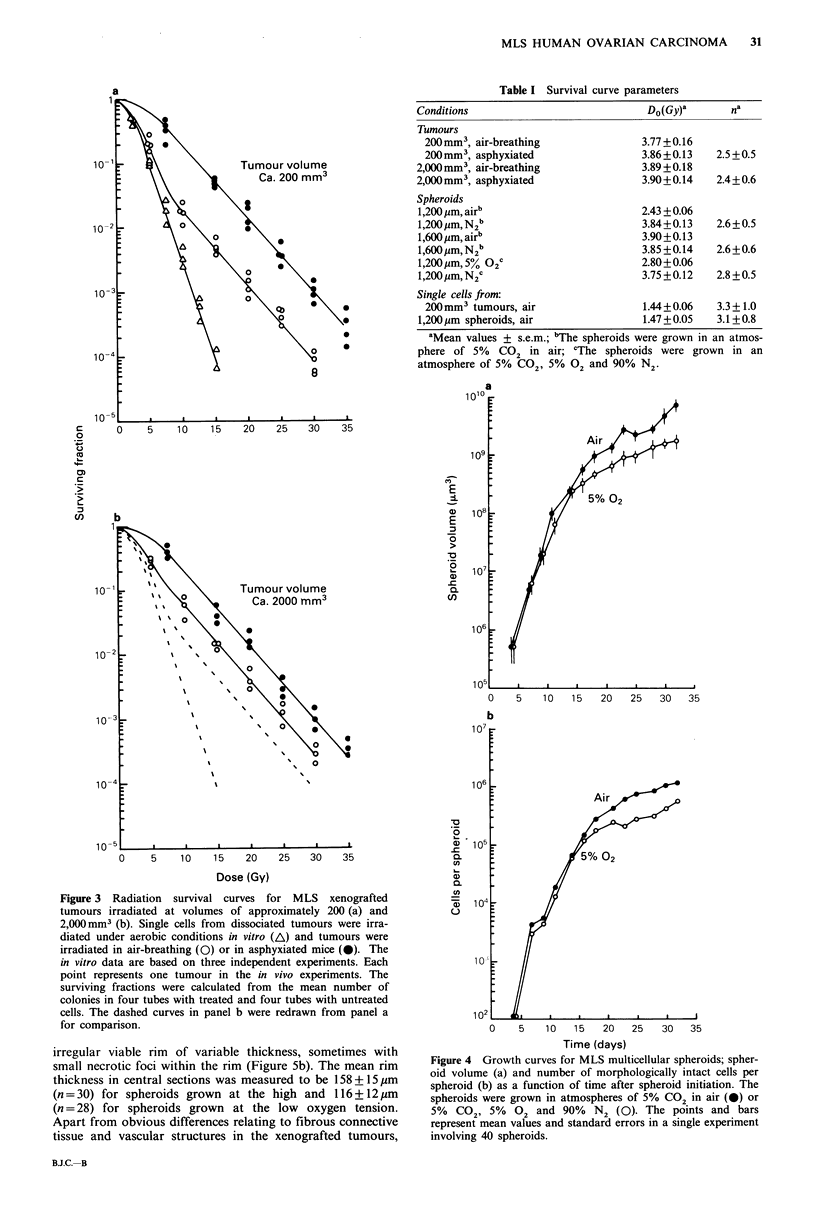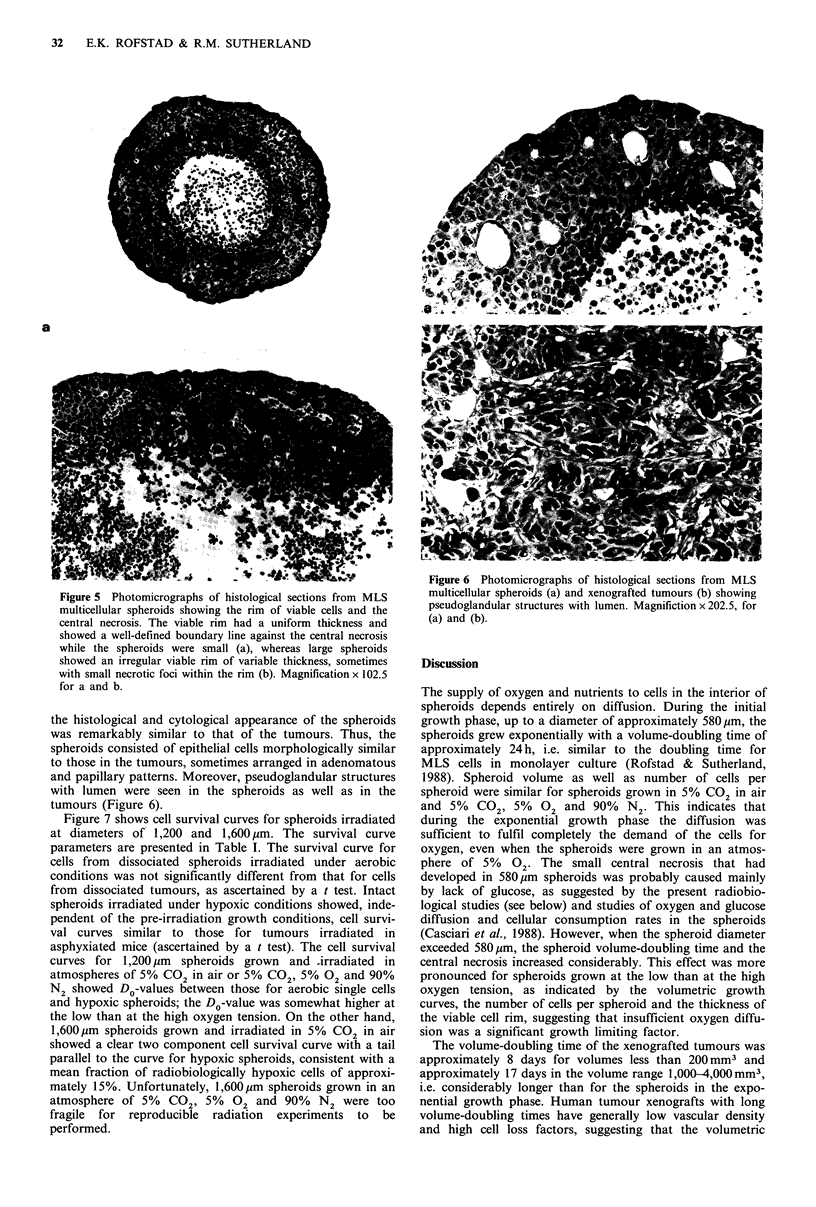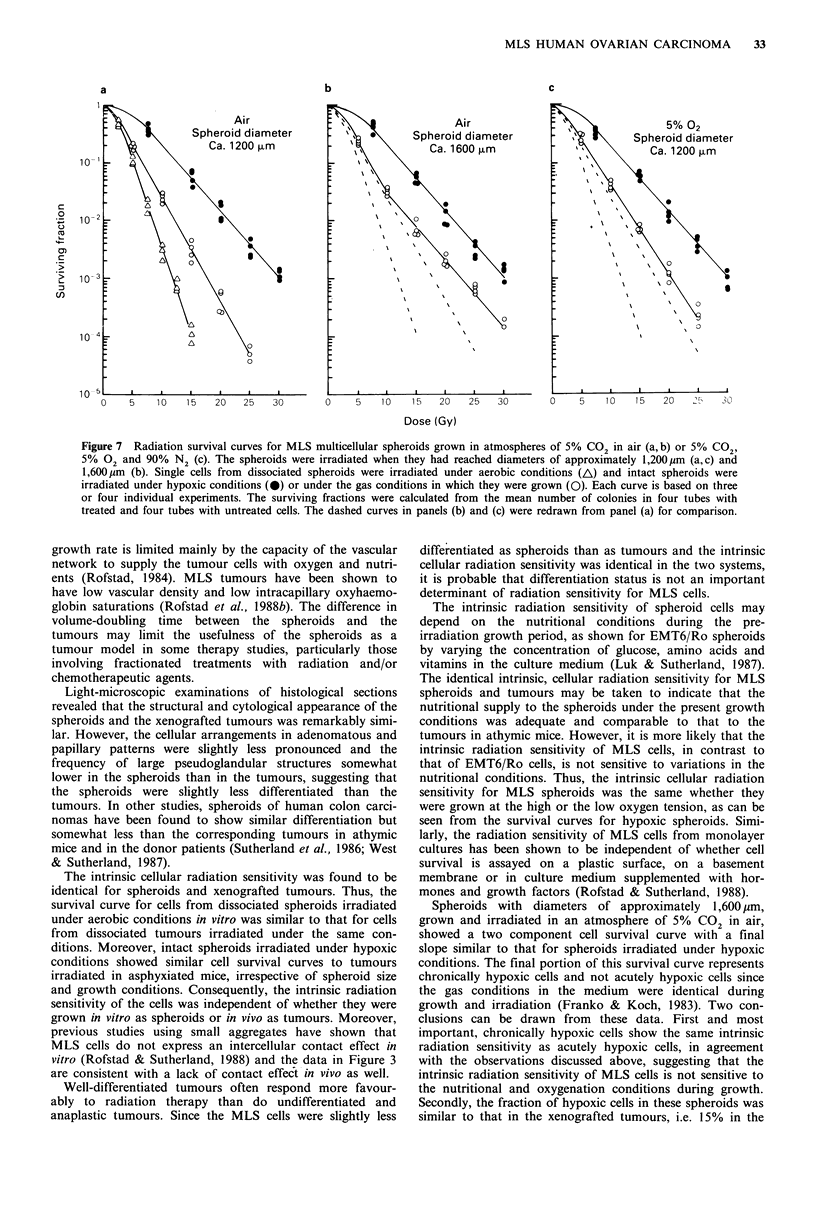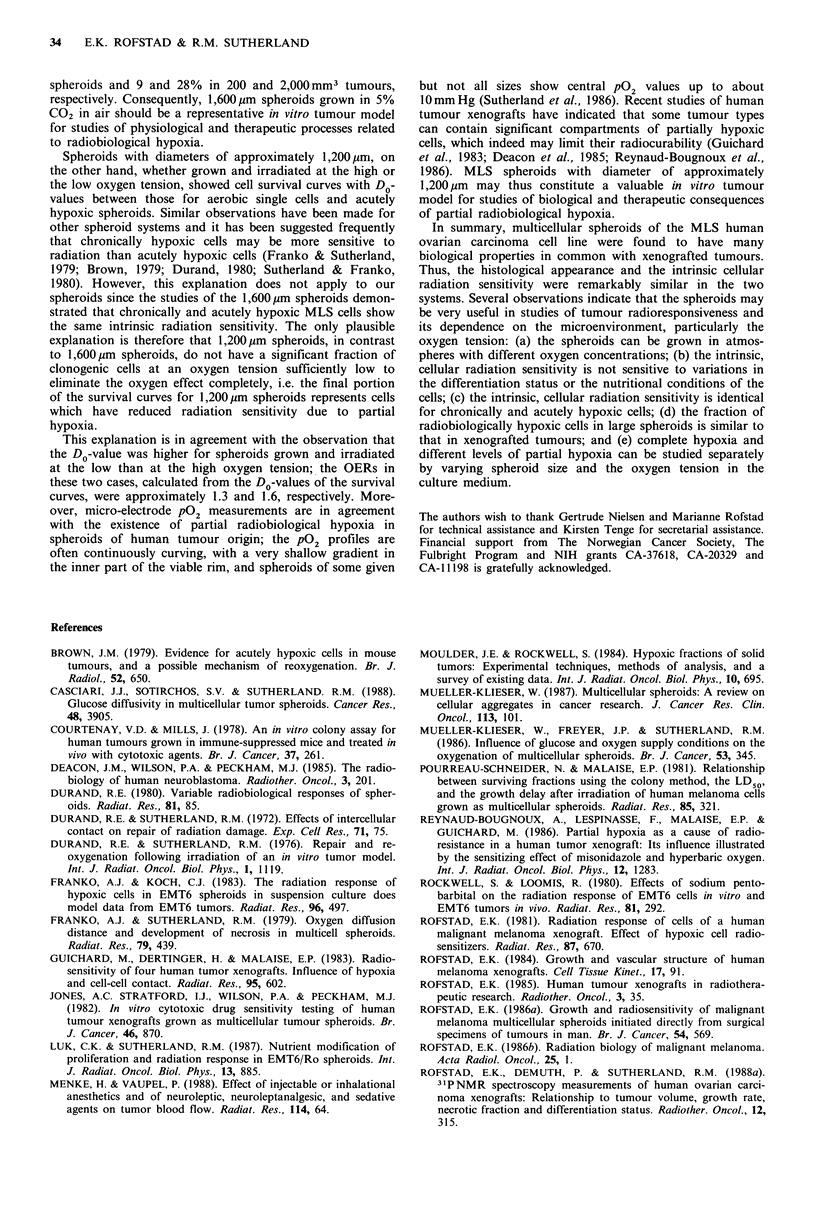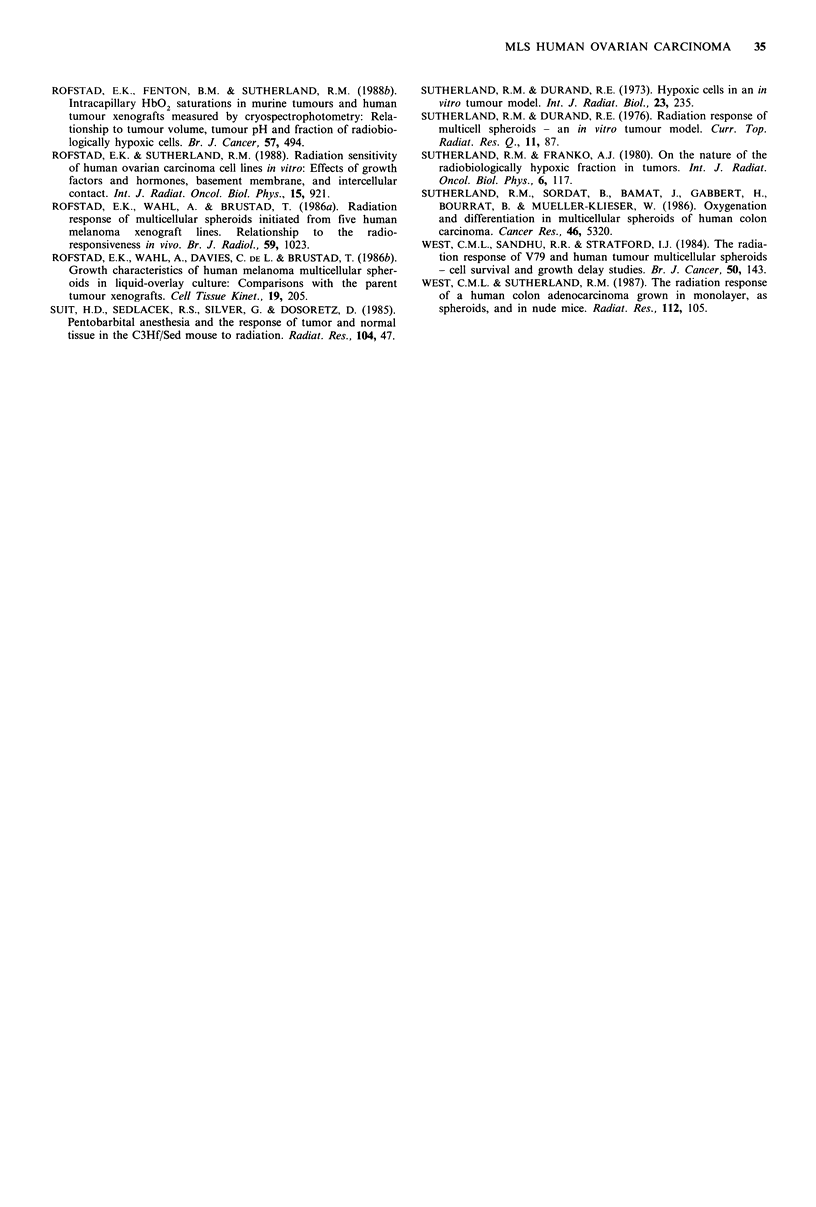# Growth and radiation sensitivity of the MLS human ovarian carcinoma cell line grown as multicellular spheroids and xenografted tumours.

**DOI:** 10.1038/bjc.1989.7

**Published:** 1989-01

**Authors:** E. K. Rofstad, R. M. Sutherland

**Affiliations:** Experimental Therapeutics Division, University of Rochester Cancer Center, New York.

## Abstract

**Images:**


Br. J. Cancer (1989), 59, 28–35 © The Macmillan Press Ltd., 1989

### Growth and radiation sensitivity of the MLS human ovarian carcinoma cell line grown as multicellular spheroids and xenografted tumours

E.K. Rofstad & R.M. Sutherland

*Experimental Therapeutics Division and Departments of Radiation Oncology and Biophysics, University of Rochester Cancer Center, Rochester, New York, USA.*

**Summary** The growth characteristics and the radiation sensitivity of multicellular spheroids of the MLS human ovarian carcinoma cell line grown in spinner culture in atmospheres of 5% $CO_2$ in air or 5% $CO_2$, 5% $O_2$ and 90% $N_2$ were studied and compared to that of MLS xenografted tumours. The spheroids grew exponentially with a volume-doubling time of approximately 24 h up to a diameter of approximately 580 $\mu$m and then the growth rate tapered off, more for spheroids grown at the low than at the high oxygen tension. Thirty days after initiation the spheroid diameters were approximately 1,500 $\mu$m at the low and 2,100 $\mu$m at the high oxygen tension. The tumour volume-doubling times were approximately 8 days ($V<200\,mm^3$) and 17 days ($V=1{,}000$–$4{,}000\,mm^3$). The histological appearance of the spheroids and the tumours was remarkably similar; both developed large central necrosis and both were composed of epithelial cells and showed pseudoglandular structures with lumen. The spheroids were slightly less differentiated than the tumours. The intrinsic, cellular radiation sensitivity was independent of whether the cells were grown *in vitro* as spheroids or *in vivo* as tumours, as revealed by irradiating single cells from dissociated spheroids and tumours under aerobic conditions and intact spheroids and tumours under hypoxic conditions. Studies of 1,600 $\mu$m spheroids grown in 5% $CO_2$ in air showed that the intrinsic radiation sensitivity of the chronically hypoxic cells was the same as that of acutely hypoxic cells. The fraction of radiobiologically hypoxic cells under these conditions was approximately 15% and similar to those of 9% ($V=200\,mm^3$) and 28% ($V=2{,}000\,mm^3$) found for the tumours. Spheroids with diameter of 1,200 $\mu$m did not show survival curves parallel to those for acutely hypoxic cells, i.e. they did not contain a measurable fraction of clonogenic cells at complete radiobiological hypoxia. The final portion of their survival curves represented partially hypoxic cells; the OERs were 1.6 and 1.3 for spheroids grown at the high and the low oxygen tension, respectively. The considerable similarity between the spheroids and the tumours suggests that MLS spheroids constitute a valuable *in vitro* model for studies of human tumour radiation biology and related physiological processes. MLS spheroids may be particularly useful in studies of therapeutic consequences of partial radiobiological hypoxia since complete hypoxia and different levels of partial hypoxia can be studied separately by varying spheroid size and the oxygen tension in the culture medium.

Multicellular spheroids are an *in vitro* tumour model system which is currently used in many aspects of cancer research, including studies of cell proliferation and differentiation, invasion and metastasis, host versus tumour reactions and tumour therapy (see Mueller-Klieser (1987) for a review). The model has several biological properties making it particularly interesting in studies of tumour radiation biology and experimental radiation therapy (Sutherland & Durand, 1976). Thus, large spheroids have diffusion gradients for oxygen, glucose and other nutrients, which result in necrotic areas, quiescent cells, radiobiologically hypoxic cells and cells at reduced pH (Sutherland & Durand, 1973, 1976). Moreover, important radiobiological phenomena, such as contact effect, repair processes and reoxygenation, have been demonstrated (Durand & Sutherland, 1972, 1976; Sutherland & Durand, 1973). Most spheroids used in radiobiological studies have been initiated from rodent cell lines, usually V79 Chinese hamster cells or EMT6 mouse mammary tumour cells. However, human tumour spheroids have been used more frequently in recent years as cell culturing techniques have been improved, and the results from human tumour spheroids seem to confirm those from rodent spheroids (Pourreau-Schneider & Malaise, 1981; Jones *et al.*, 1982; West *et al.*, 1984; Rofstad, 1986a).

Methods for xenografting human tumours into immune-deficient animals have been developed and made it possible to compare the radiation biology of human tumour spheroids with that of tumours of the same cells (e.g. Rofstad, 1985). Thus, Rofstad *et al.* (1986*a,b*) studied five human melanomas and found that the radiation sensitivity of small spheroids, i.e. spheroids that had not developed radiobiological hypoxia or necrosis, was similar to that of the corresponding melanoma xenografted tumours, whether single cell survival or growth delay was used as endpoint. Similarly, there was good agreement between spheroids and tumours regarding expression of the intercellular contact effect; melanomas that showed a contact effect as tumours *in vivo* were found to show a contact effect as small spheroids *in vitro* as well and *vice versa* (Rofstad, 1986*b*; Rofstad *et al.*, 1986*a*). Comparisons of the radiation biology of human tumour spheroids and the corresponding xenografted tumours using large spheroids with heterogeneous oxygenation and radiobiologically hypoxic cells have not been reported so far, with the exception of one study involving one cell line. West & Sutherland (1987) studied the WiDr human colon adenocarcinoma and concluded that the spheroids appeared to model accurately the cellular radiation sensitivity of the tumours. Moreover, using 1,200 $\mu$m spheroids grown and irradiated in an atmosphere of 3% $CO_2$ in air, they found the fraction of radiobiologically hypoxic cells to be 8%, which agreed fairly well with that of 12% found in 8–10 mm tumours (West & Sutherland, 1987).

The radiation biology of large spheroids and xenografted tumours of the MLS human ovarian carcinoma cell line is reported in the present communication; the main purpose of the work was to compare the growth, the cellular radiation sensitivity and the fraction of radiobiologically hypoxic cells in the spheroids and the tumours. Studies of rodent spheroid cultures have shown that these three parameters may all depend on the experimental conditions, the nutrient supply, the oxygen tension in the culture medium and the spheroid diameter (Durand, 1980; Franko & Koch, 1983; Mueller-Klieser *et al.*, 1986; Luk & Sutherland, 1987). Moreover, the fraction of radiobiologically hypoxic cells in tumours may vary significantly with tumour volume (Moulder & Rockwell, 1984). Consequently, spheroids and tumours of different, distinct volumes were studied in the present work. In addition, the spheroids were grown in two different

Correspondence: E.K. Rofstad, Institute for Cancer Research, The Norwegian Radium Hospital, Montebello, 0310 Oslo 3, Norway.
Received 8 May 1988; and in revised form, 8 August 1988.

atmospheres, 5% $CO_2$ in air and 5% $CO_2$, 5% $O_2$ and 90% $N_2$, the latter oxygen tension being closer to that in tissues *in vivo* than that of air usually used in tissue culture studies *in vitro*.

### Materials and methods

### *Cell line*

The MLS human ovarian carcinoma cell line was established from a serous cystadenocarcinoma of a patient admitted to the University of Rochester Cancer Center. The patient had been treated with cytoxan, adriamycin, cisplatin, 5-fluorouracil, methotrexate, tamoxifen and depoprovera before tumour cells were removed for establishment of a cell line *in vitro* (Rofstad & Sutherland, 1988). The cell line was maintained as a monolayer cell culture in α-minimum essential medium with 5.5 mM glucose (Gibco Laboratories, Grand Island, NY) containing 10% fetal calf serum (J.R. Scientific, Woodland, CA) and was subcultured routinely once a week. The medium was supplemented with 100 IU ml$^{-1}$ penicillin (ICN Nutritional Biochemicals, Cleveland, OH) and 0.1 mg ml$^{-1}$ streptomycin (Gibco Laboratories, Grand Island, NY), and 2 mM L-glutamine (Eastman Kodak Company, Rochester, NY) was replenished in medium stored for longer than two weeks. The cell cultures were maintained in a humidified atmosphere of 5% $CO_2$ in air. The doubling time of the cells was approximately 24 h under these conditions (Rofstad & Sutherland, 1988). The cells used in the present experiments had been grown for more than 100 passages *in vitro*, i.e. the experiments were performed with an established cell line. The ovarian origin of the cell line was confirmed using ovarian carcinoma associated monoclonal antibodies. The cell line was routinely monitored for *Mycoplasma* contamination using both the Hoechst fluorescence and mycotrim methods. The trypsin (Cooper, West Chester, PA) used for routine subculturing and in spheroid experiments (see below) was lyophilised at 202 IU mg$^{-1}$ and prepared in 0.02% ethylenediaminetetra-acetic acid (EDTA) combined in phosphate buffered saline (PBS) (pH = 7.3).

### *Spheroid cultures*

Multicellular spheroids were initiated by seeding approximately $1.0 \times 10^6$ cells in 30 ml culture medium in 75 cm$^2$ plastic tissue culture flasks (Costar, Cambridge, MA) coated with a thin layer (3 ml per flask) of 1% agar (Difco Laboratories, Detroit, MI). The flasks were then agitated gently for 30 min and small aggregates, 10–20 cells per aggregate, were formed. The aggregates were allowed to grow in these culture flasks for 4 days and at that time small spheroids, approximately 100 μm in diameter, had developed. Then the spheroids were filtered through 90 and 100 μm nylon screens to obtain a homogenous spheroid population. Approximately 2,000 spheroids were seeded into 100 mm diameter spinner flasks (Bellco Glass, Vineland, NJ) containing 300 ml culture medium. The flasks were placed on magnetic stirrers set at 110 r.p.m. in a 37°C room and gassed daily with 5% $CO_2$ in air or 5% $CO_2$, 5% $O_2$ and 90% $N_2$ gas mixtures. The culture medium was replaced three times per week, and when the spheroids had reached approximately 300, 500 and 1,000 μm in diameter the number of spheroids per flask was reduced to 1,000, 300 and 100, respectively. At 500 μm in diameter, the spheroid culture spinning rate was increased to 190 r.p.m. Spheroid volumes were calculated as $(\pi/6) \times d_1 \times d_2{}^2$ where $d_1$ and $d_2$ are the longest and the shortest, respectively, of two orthogonal spheroid diameters measured with an ocular micrometer in an inverted phase-contrast microscope. The spheroids were dissociated by trypsinisation at 37°C for 30 min and gentle agitation, and the number of morphologically intact cells per spheroid was determined using a haemocytometer. Histological sections were prepared from the spheroids as described elsewhere (Sutherland *et al.*, 1986).

### *Xenografted tumours*

The MLS tumour line was initiated by inoculating $1 \times 10^7$ cells subcutaneously into the flank of BALB/c athymic mice (Life Sciences, St Petersburg, FL) and maintained by serial, subcutaneous transplantation of tumour fragments, approximately 2 × 2 × 2 mm in size (Rofstad *et al.*, 1988*a*). Subcutaneous tumours in passages 4 and 5 growing in the left flank of 8–10-week-old female athymic mice kept in a humidified, aseptic environment were used in the present work. Tumour volume was measured with callipers. Two orthogonal diameters (length and width) were recorded, and tumour volume was calculated as $V = \frac{1}{2}ab^2$ where $a$ and $b$ are the longest and the shortest diameter, respectively. Single cell suspensions were prepared from the tumours by incubation at 37°C for 30 min in an enzyme mixture containing 0.025% collagenase I (Sigma Chemical, St Louis, MO), 0.025% pronase (Calbiochem, San Diego, CA) and 0.02% DNase (Sigma Chemical, St Louis, MO). Adequate cell yields were obtained routinely in order to measure cell survival levels down to $1 \times 10^{-4}$. Histological sections were prepared using standard procedures and haematoxylin and eosin staining.

### *Irradiation procedures*

Spheroids were irradiated at a dose rate of 1.75 Gy min$^{-1}$ and single cells and tumours at a dose rate of 5.2 Gy min$^{-1}$ using a $^{137}$Cs γ-ray source. The radiation sensitivity of single cells was not significantly different at these two dose rates.

Single cells from dissociated spheroids and tumours were resuspended in culture medium at a concentration of $1 \times 10^5$ cells ml$^{-1}$. Aliquots of 20 ml of the cell suspensions were transferred to 75 cm$^2$ tissue culture flasks (Costar, Cambridge, MA), gassed with 5% $CO_2$ in air and irradiated.

Spheroids of the appropriate size were hand-picked from the spinner flasks in which they were grown and transferred to new spinner flasks with fresh medium for irradiation, 25 spheroids per flask and one flask per radiation dose. The new flasks were gassed with the correct gas mixture, 5% $CO_2$ in air or 5% $CO_2$, 5% $O_2$ and 90% $N_2$, sealed and incubated at 37°C on magnetic stirrers for 48 h before irradiation. During irradiation the spinner flasks were kept at 37°C by a water bath and the stir rate was 190 r.p.m. as before irradiation. Hypoxic conditions were obtained by gassing the spinner flasks with 5% $CO_2$ plus 95% $N_2$ at a flow rate of 400 ml min$^{-1}$ for 2 h at 37°C immediately before irradiation.

Tumours having a volume of approximately 200 and 2,000 mm$^3$ were irradiated *in vivo* in mice anaesthetised with sodium pentobarbitone, 0.09 mg per g body weight. The body core temperature of the mice was kept at 37–38°C during irradiation by using a heating pad with circulating water. The cellular radiation sensitivity as well as the fraction of radiobiologically hypoxic cells in the tumours are unlikely to be significantly influenced by the sodium pentobarbitone anaesthesia under these conditions (Rockwell & Loomis, 1980; Suit *et al.*, 1985; Menke & Vaupel, 1988). Mice were asphyxiated (cervical dislocation) 15 min before irradiation to obtain hypoxic conditions.

### *Colony assay*

Single cell suspensions were prepared from the spheroids and tumours immediately after irradiation as described above and cell survival was measured using an *in vitro* soft agar colony assay similar to that developed by Courtenay & Mills (1978). The soft agar was prepared from powdered agar (Difco Laboratories, Detroit, MI) and culture medium supplemented with 20% fetal calf serum and antibiotics. Rat erythrocytes and tumour cells were added as described previously (Rofstad, 1981). Aliquots of 1 ml of soft agar were seeded in Falcon 2057 plastic tubes (Becton Dickinson,

Lincoln Park, NJ). The number of tumour cells seeded per tube was in the range $1 \times 10^3$ to $1 \times 10^6$. The cells were then incubated at 37°C for 5 weeks in an atmosphere of 5% $O_2$, 5% $CO_2$ and 90% $N_2$. Culture medium (2 ml) was added on the top of the agar 5 days after seeding and then changed weekly. Colonies were counted using a stereomicroscope. Cells giving rise to colonies larger than 50 cells were scored as surviving. The number of colonies scored per tube was usually in the range 50–200. A minimum of five colonies per tube was scored for tumours given the highest radiation dose. The plating efficiency was 40–60% for morphologically intact cells from spheroids. Cell suspensions from tumours consisted of approximately 60% human ovarian carcinoma cells and approximately 40% host cells. The plating efficiency was 5–10%, calculated from the number of colonies formed and the number of morphologically intact human ovarian carcinoma cells seeded. Heavily irradiated feeder cells did not enhance the plating efficiency.

*Data analysis*

Survival curves were fitted to the data by least-squares linear regression analysis. The analysis was based on individual surviving fractions measured in the dose ranges 5–15 Gy (single cells), 5–25 Gy (1,200 $\mu$m spheroids, air and 5% $O_2$), 10–30 Gy (1,600 $\mu$m spheroids, air; tumours, air-breathing mice) and 7.5–35 Gy (spheroids and tumours, hypoxia).

### Results

The volume-doubling time of the MLS tumours was approximately 8 days for volumes less than 200 mm³ and approximately 17 days in the volume range 1,000–4,000 mm³. A volume of 4,000 mm³ was reached about 90 days post-implantation (Figure 1). Histological investigations showed that the tumours developed large central necrotic areas; the area fraction of necrosis was 30–40% for tumours less than 200 mm³ and 50–70% for tumours larger than 1,000 mm³. The areas with viable tissue consisted of multiple lobular masses composed of epithelial cells arranged in slightly adenomatous and papillary patterns. Minor clusters of fibrous connective tissue were also seen, but mainly in the periphery of the tumours. The tumours were classified as poorly differentiated (Figure 2).

Radiation survival curves for cells from dissociated tumours irradiated *in vitro* and tumours irradiated *in vivo*

**Figure 1** Growth curve for MLS xenografted tumours. The points and the bars represent mean values and standard errors in a single experiment involving 16 individual tumours.

**Figure 2** Photomicrographs of histological sections from MLS xenografted tumours showing necrosis and viable tissue. The viable tissue consisted of multiple lobular masses composed of epithelial cells arranged in slightly adenomatous and papillary patterns. The MLS tumour line was classified as poorly differentiated. Magnification × 102.5 (a) and 202.5 (b).

and assayed *in vitro* are presented in Figure 3. The $D_0$-value for the hypoxic cells was not significantly different for tumours irradiated in air-breathing and asphyxiated mice (ascertained by a *t* test), independent of tumour volume, and 2.6–2.7 times larger than that for tumour cells irradiated under aerobic conditions *in vitro* (Table I). The mean fraction of radiobiologically hypoxic cells in the tumours, determined from the vertical displacement of the survival curves pertaining to air-breathing and asphyxiated mice, was found to be approximately 9% at a volume of 200 mm³ and approximately 28% at a volume of 2,000 mm³.

The volumetric growth of the MLS spheroids was exponential up to a volume of approximately $1 \times 10^8$ $\mu m^3$ (diameter of 580 $\mu$m) and then the growth rate tapered off, more for spheroids grown in 5% $CO_2$, 5% $O_2$ and 90% $N_2$ than in 5% $CO_2$ in air (Figure 4a). The volume-doubling time during the exponential growth phase was approximately 24 h, irrespective of the oxygen tension in the culture medium. Thirty days after initiation the spheroid diameters were approximately 1,500 $\mu$m at the low and approximately 2,100 $\mu$m at the high oxygen tension. Similar growth curves were found when the number of morphologically intact cells per spheroid was plotted against time (Figure 4b), i.e. the volume measurements reflected the number of morphologically intact cells in the spheroids.

Histologically, the spheroids consisted of a rim of viable cells surrounding a necrotic centre. When the spheroids were small (diameter of 500–600 $\mu$m), the viable rim had a uniform thickness and showed a well-defined boundary line against the central necrosis (Figure 5a). Large spheroids (diameter of 1,400–2,100 $\mu$m) on the other hand showed an

**Figure 3** Radiation survival curves for MLS xenografted tumours irradiated at volumes of approximately 200 (a) and 2,000 mm³ (b). Single cells from dissociated tumours were irradiated under aerobic conditions *in vitro* (△) and tumours were irradiated in air-breathing (○) or in asphyxiated mice (●). The *in vitro* data are based on three independent experiments. Each point represents one tumour in the *in vivo* experiments. The surviving fractions were calculated from the mean number of colonies in four tubes with treated and four tubes with untreated cells. The dashed curves in panel b were redrawn from panel a for comparison.

**Table I** Survival curve parameters

| *Conditions* | $D_0(Gy)$[a] | $n$[a] |
|---|---|---|
| *Tumours* | | |
| 200 mm³, air-breathing | 3.77±0.16 | |
| 200 mm³, asphyxiated | 3.86±0.13 | 2.5±0.5 |
| 2,000 mm³, air-breathing | 3.89±0.18 | |
| 2,000 mm³, asphyxiated | 3.90±0.14 | 2.4±0.6 |
| *Spheroids* | | |
| 1,200 μm, air[b] | 2.43±0.06 | |
| 1,200 μm, $N_2$[b] | 3.84±0.13 | 2.6±0.5 |
| 1,600 μm, air[b] | 3.90±0.13 | |
| 1,600 μm, $N_2$[b] | 3.85±0.14 | 2.6±0.6 |
| 1,200 μm, 5% $O_2$[c] | 2.80±0.06 | |
| 1,200 μm, $N_2$[c] | 3.75±0.12 | 2.8±0.5 |
| *Single cells from:* | | |
| 200 mm³ tumours, air | 1.44±0.06 | 3.3±1.0 |
| 1,200 μm spheroids, air | 1.47±0.05 | 3.1±0.8 |

[a]Mean values ± s.e.m.; [b]The spheroids were grown in an atmosphere of 5% $CO_2$ in air; [c]The spheroids were grown in an atmosphere of 5% $CO_2$, 5% $O_2$ and 90% $N_2$.

**Figure 4** Growth curves for MLS multicellular spheroids; spheroid volume (a) and number of morphologically intact cells per spheroid (b) as a function of time after spheroid initiation. The spheroids were grown in atmospheres of 5% $CO_2$ in air (●) or 5% $CO_2$, 5% $O_2$ and 90% $N_2$ (○). The points and bars represent mean values and standard errors in a single experiment involving 40 spheroids.

irregular viable rim of variable thickness, sometimes with small necrotic foci within the rim (Figure 5b). The mean rim thickness in central sections was measured to be 158±15 μm ($n=30$) for spheroids grown at the high and 116±12 μm ($n=28$) for spheroids grown at the low oxygen tension. Apart from obvious differences relating to fibrous connective tissue and vascular structures in the xenografted tumours,

**Figure 5** Photomicrographs of histological sections from MLS multicellular spheroids showing the rim of viable cells and the central necrosis. The viable rim had a uniform thickness and showed a well-defined boundary line against the central necrosis while the spheroids were small (a), whereas large spheroids showed an irregular viable rim of variable thickness, sometimes with small necrotic foci within the rim (b). Magnification × 102.5 for a and b.

the histological and cytological appearance of the spheroids was remarkably similar to that of the tumours. Thus, the spheroids consisted of epithelial cells morphologically similar to those in the tumours, sometimes arranged in adenomatous and papillary patterns. Moreover, pseudoglandular structures with lumen were seen in the spheroids as well as in the tumours (Figure 6).

Figure 7 shows cell survival curves for spheroids irradiated at diameters of 1,200 and 1,600 $\mu$m. The survival curve parameters are presented in Table I. The survival curve for cells from dissociated spheroids irradiated under aerobic conditions was not significantly different from that for cells from dissociated tumours, as ascertained by a *t* test. Intact spheroids irradiated under hypoxic conditions showed, independent of the pre-irradiation growth conditions, cell survival curves similar to those for tumours irradiated in asphyxiated mice (ascertained by a *t* test). The cell survival curves for 1,200 $\mu$m spheroids grown and irradiated in atmospheres of 5% $CO_2$ in air or 5% $CO_2$, 5% $O_2$ and 90% $N_2$ showed $D_0$-values between those for aerobic single cells and hypoxic spheroids; the $D_0$-value was somewhat higher at the low than at the high oxygen tension. On the other hand, 1,600 $\mu$m spheroids grown and irradiated in 5% $CO_2$ in air showed a clear two component cell survival curve with a tail parallel to the curve for hypoxic spheroids, consistent with a mean fraction of radiobiologically hypoxic cells of approximately 15%. Unfortunately, 1,600 $\mu$m spheroids grown in an atmosphere of 5% $CO_2$, 5% $O_2$ and 90% $N_2$ were too fragile for reproducible radiation experiments to be performed.

**Figure 6** Photomicrographs of histological sections from MLS multicellular spheroids (a) and xenografted tumours (b) showing pseudoglandular structures with lumen. Magnifiction × 202.5, for (a) and (b).

### Discussion

The supply of oxygen and nutrients to cells in the interior of spheroids depends entirely on diffusion. During the initial growth phase, up to a diameter of approximately 580 $\mu$m, the spheroids grew exponentially with a volume-doubling time of approximately 24 h, i.e. similar to the doubling time for MLS cells in monolayer culture (Rofstad & Sutherland, 1988). Spheroid volume as well as number of cells per spheroid were similar for spheroids grown in 5% $CO_2$ in air and 5% $CO_2$, 5% $O_2$ and 90% $N_2$. This indicates that during the exponential growth phase the diffusion was sufficient to fulfil completely the demand of the cells for oxygen, even when the spheroids were grown in an atmosphere of 5% $O_2$. The small central necrosis that had developed in 580 $\mu$m spheroids was probably caused mainly by lack of glucose, as suggested by the present radiobiological studies (see below) and studies of oxygen and glucose diffusion and cellular consumption rates in the spheroids (Casciari *et al.*, 1988). However, when the spheroid diameter exceeded 580 $\mu$m, the spheroid volume-doubling time and the central necrosis increased considerably. This effect was more pronounced for spheroids grown at the low than at the high oxygen tension, as indicated by the volumetric growth curves, the number of cells per spheroid and the thickness of the viable cell rim, suggesting that insufficient oxygen diffusion was a significant growth limiting factor.

The volume-doubling time of the xenografted tumours was approximately 8 days for volumes less than 200 $mm^3$ and approximately 17 days in the volume range 1,000–4,000 $mm^3$, i.e. considerably longer than for the spheroids in the exponential growth phase. Human tumour xenografts with long volume-doubling times have generally low vascular density and high cell loss factors, suggesting that the volumetric

**Figure 7** Radiation survival curves for MLS multicellular spheroids grown in atmospheres of 5% $CO_2$ in air (a, b) or 5% $CO_2$, 5% $O_2$ and 90% $N_2$ (c). The spheroids were irradiated when they had reached diameters of approximately 1,200 μm (a, c) and 1,600 μm (b). Single cells from dissociated spheroids were irradiated under aerobic conditions (△) and intact spheroids were irradiated under hypoxic conditions (●) or under the gas conditions in which they were grown (○). Each curve is based on three or four individual experiments. The surviving fractions were calculated from the mean number of colonies in four tubes with treated and four tubes with untreated cells. The dashed curves in panels (b) and (c) were redrawn from panel (a) for comparison.

growth rate is limited mainly by the capacity of the vascular network to supply the tumour cells with oxygen and nutrients (Rofstad, 1984). MLS tumours have been shown to have low vascular density and low intracapillary oxyhaemoglobin saturations (Rofstad *et al.*, 1988*b*). The difference in volume-doubling time between the spheroids and the tumours may limit the usefulness of the spheroids as a tumour model in some therapy studies, particularly those involving fractionated treatments with radiation and/or chemotherapeutic agents.

Light-microscopic examinations of histological sections revealed that the structural and cytological appearance of the spheroids and the xenografted tumours was remarkably similar. However, the cellular arrangements in adenomatous and papillary patterns were slightly less pronounced and the frequency of large pseudoglandular structures somewhat lower in the spheroids than in the tumours, suggesting that the spheroids were slightly less differentiated than the tumours. In other studies, spheroids of human colon carcinomas have been found to show similar differentiation but somewhat less than the corresponding tumours in athymic mice and in the donor patients (Sutherland *et al.*, 1986; West & Sutherland, 1987).

The intrinsic cellular radiation sensitivity was found to be identical for spheroids and xenografted tumours. Thus, the survival curve for cells from dissociated spheroids irradiated under aerobic conditions *in vitro* was similar to that for cells from dissociated tumours irradiated under the same conditions. Moreover, intact spheroids irradiated under hypoxic conditions showed similar cell survival curves to tumours irradiated in asphyxiated mice, irrespective of spheroid size and growth conditions. Consequently, the intrinsic radiation sensitivity of the cells was independent of whether they were grown *in vitro* as spheroids or *in vivo* as tumours. Moreover, previous studies using small aggregates have shown that MLS cells do not express an intercellular contact effect *in vitro* (Rofstad & Sutherland, 1988) and the data in Figure 3 are consistent with a lack of contact effect *in vivo* as well.

Well-differentiated tumours often respond more favourably to radiation therapy than do undifferentiated and anaplastic tumours. Since the MLS cells were slightly less differentiated as spheroids than as tumours and the intrinsic cellular radiation sensitivity was identical in the two systems, it is probable that differentiation status is not an important determinant of radiation sensitivity for MLS cells.

The intrinsic radiation sensitivity of spheroid cells may depend on the nutritional conditions during the pre-irradiation growth period, as shown for EMT6/Ro spheroids by varying the concentration of glucose, amino acids and vitamins in the culture medium (Luk & Sutherland, 1987). The identical intrinsic, cellular radiation sensitivity for MLS spheroids and tumours may be taken to indicate that the nutritional supply to the spheroids under the present growth conditions was adequate and comparable to that to the tumours in athymic mice. However, it is more likely that the intrinsic radiation sensitivity of MLS cells, in contrast to that of EMT6/Ro cells, is not sensitive to variations in the nutritional conditions. Thus, the intrinsic cellular radiation sensitivity for MLS spheroids was the same whether they were grown at the high or the low oxygen tension, as can be seen from the survival curves for hypoxic spheroids. Similarly, the radiation sensitivity of MLS cells from monolayer cultures has been shown to be independent of whether cell survival is assayed on a plastic surface, on a basement membrane or in culture medium supplemented with hormones and growth factors (Rofstad & Sutherland, 1988).

Spheroids with diameters of approximately 1,600 μm, grown and irradiated in an atmosphere of 5% $CO_2$ in air, showed a two component cell survival curve with a final slope similar to that for spheroids irradiated under hypoxic conditions. The final portion of this survival curve represents chronically hypoxic cells and not acutely hypoxic cells since the gas conditions in the medium were identical during growth and irradiation (Franko & Koch, 1983). Two conclusions can be drawn from these data. First and most important, chronically hypoxic cells show the same intrinsic radiation sensitivity as acutely hypoxic cells, in agreement with the observations discussed above, suggesting that the intrinsic radiation sensitivity of MLS cells is not sensitive to the nutritional and oxygenation conditions during growth. Secondly, the fraction of hypoxic cells in these spheroids was similar to that in the xenografted tumours, i.e. 15% in the spheroids and 9 and 28% in 200 and 2,000 $mm^3$ tumours, respectively. Consequently, 1,600 $\mu m$ spheroids grown in 5% $CO_2$ in air should be a representative *in vitro* tumour model for studies of physiological and therapeutic processes related to radiobiological hypoxia.

Spheroids with diameters of approximately 1,200 $\mu m$, on the other hand, whether grown and irradiated at the high or the low oxygen tension, showed cell survival curves with $D_0$-values between those for aerobic single cells and acutely hypoxic spheroids. Similar observations have been made for other spheroid systems and it has been suggested frequently that chronically hypoxic cells may be more sensitive to radiation than acutely hypoxic cells (Franko & Sutherland, 1979; Brown, 1979; Durand, 1980; Sutherland & Franko, 1980). However, this explanation does not apply to our spheroids since the studies of the 1,600 $\mu m$ spheroids demonstrated that chronically and acutely hypoxic MLS cells show the same intrinsic radiation sensitivity. The only plausible explanation is therefore that 1,200 $\mu m$ spheroids, in contrast to 1,600 $\mu m$ spheroids, do not have a significant fraction of clonogenic cells at an oxygen tension sufficiently low to eliminate the oxygen effect completely, i.e. the final portion of the survival curves for 1,200 $\mu m$ spheroids represents cells which have reduced radiation sensitivity due to partial hypoxia.

This explanation is in agreement with the observation that the $D_0$-value was higher for spheroids grown and irradiated at the low than at the high oxygen tension; the OERs in these two cases, calculated from the $D_0$-values of the survival curves, were approximately 1.3 and 1.6, respectively. Moreover, micro-electrode $pO_2$ measurements are in agreement with the existence of partial radiobiological hypoxia in spheroids of human tumour origin; the $pO_2$ profiles are often continuously curving, with a very shallow gradient in the inner part of the viable rim, and spheroids of some given but not all sizes show central $pO_2$ values up to about 10 mmHg (Sutherland *et al.*, 1986). Recent studies of human tumour xenografts have indicated that some tumour types can contain significant compartments of partially hypoxic cells, which indeed may limit their radiocurability (Guichard *et al.*, 1983; Deacon *et al.*, 1985; Reynaud-Bougnoux *et al.*, 1986). MLS spheroids with diameter of approximately 1,200 $\mu m$ may thus constitute a valuable *in vitro* tumour model for studies of biological and therapeutic consequences of partial radiobiological hypoxia.

In summary, multicellular spheroids of the MLS human ovarian carcinoma cell line were found to have many biological properties in common with xenografted tumours. Thus, the histological appearance and the intrinsic cellular radiation sensitivity were remarkably similar in the two systems. Several observations indicate that the spheroids may be very useful in studies of tumour radioresponsiveness and its dependence on the microenvironment, particularly the oxygen tension: (a) the spheroids can be grown in atmospheres with different oxygen concentrations; (b) the intrinsic, cellular radiation sensitivity is not sensitive to variations in the differentiation status or the nutritional conditions of the cells; (c) the intrinsic, cellular radiation sensitivity is identical for chronically and acutely hypoxic cells; (d) the fraction of radiobiologically hypoxic cells in large spheroids is similar to that in xenografted tumours; and (e) complete hypoxia and different levels of partial hypoxia can be studied separately by varying spheroid size and the oxygen tension in the culture medium.

The authors wish to thank Gertrude Nielsen and Marianne Rofstad for technical assistance and Kirsten Tenge for secretarial assistance. Financial support from The Norwegian Cancer Society, The Fulbright Program and NIH grants CA-37618, CA-20329 and CA-11198 is gratefully acknowledged.

### References

BROWN, J.M. (1979). Evidence for acutely hypoxic cells in mouse tumours, and a possible mechanism of reoxygenation. *Br. J. Radiol.*, **52**, 650.

CASCIARI, J.J., SOTIRCHOS, S.V. & SUTHERLAND. R.M. (1988). Glucose diffusivity in multicellular tumor spheroids. *Cancer Res.*, **48**, 3905.

COURTENAY, V.D. & MILLS, J. (1978). An *in vitro* colony assay for human tumours grown in immune-suppressed mice and treated *in vivo* with cytotoxic agents. *Br. J. Cancer*, **37**, 261.

DEACON, J.M., WILSON, P.A. & PECKHAM, M.J. (1985). The radiobiology of human neuroblastoma. *Radiother. Oncol.*, **3**, 201.

DURAND, R.E. (1980). Variable radiobiological responses of spheroids. *Radiat. Res.*, **81**, 85.

DURAND, R.E. & SUTHERLAND, R.M. (1972). Effects of intercellular contact on repair of radiation damage. *Exp. Cell Res.*, **71**, 75.

DURAND, R.E. & SUTHERLAND, R.M. (1976). Repair and reoxygenation following irradiation of an *in vitro* tumor model. *Int. J. Radiat. Oncol. Biol. Phys.*, **1**, 1119.

FRANKO, A.J. & KOCH, C.J. (1983). The radiation response of hypoxic cells in EMT6 spheroids in suspension culture does model data from EMT6 tumors. *Radiat. Res.*, **96**, 497.

FRANKO, A.J. & SUTHERLAND, R.M. (1979). Oxygen diffusion distance and development of necrosis in multicell spheroids. *Radiat. Res.*, **79**, 439.

GUICHARD, M., DERTINGER, H. & MALAISE, E.P. (1983). Radiosensitivity of four human tumor xenografts. Influence of hypoxia and cell-cell contact. *Radiat. Res.*, **95**, 602.

JONES, A.C. STRATFORD, I.J., WILSON, P.A. & PECKHAM, M.J. (1982). *In vitro* cytotoxic drug sensitivity testing of human tumour xenografts grown as multicellular tumour spheroids. *Br. J. Cancer*, **46**, 870.

LUK, C.K. & SUTHERLAND, R.M. (1987). Nutrient modification of proliferation and radiation response in EMT6/Ro spheroids. *Int. J. Radiat. Oncol. Biol. Phys.*, **13**, 885.

MENKE, H. & VAUPEL, P. (1988). Effect of injectable or inhalational anesthetics and of neuroleptic, neuroleptanalgesic, and sedative agents on tumor blood flow. *Radiat. Res.*, **114**, 64.

MOULDER, J.E. & ROCKWELL, S. (1984). Hypoxic fractions of solid tumors: Experimental techniques, methods of analysis, and a survey of existing data. *Int. J. Radiat. Oncol. Biol. Phys.*, **10**, 695.

MUELLER-KLIESER, W. (1987). Multicellular spheroids: A review on cellular aggregates in cancer research. *J. Cancer Res. Clin. Oncol.*, **113**, 101.

MUELLER-KLIESER, W., FREYER, J.P. & SUTHERLAND, R.M. (1986). Influence of glucose and oxygen supply conditions on the oxygenation of multicellular spheroids. *Br. J. Cancer*, **53**, 345.

POURREAU-SCHNEIDER, N. & MALAISE, E.P. (1981). Relationship between surviving fractions using the colony method, the $LD_{50}$, and the growth delay after irradiation of human melanoma cells grown as multicellular spheroids. *Radiat. Res.*, **85**, 321.

REYNAUD-BOUGNOUX, A., LESPINASSE, F., MALAISE, E.P. & GUICHARD, M. (1986). Partial hypoxia as a cause of radioresistance in a human tumor xenograft: Its influence illustrated by the sensitizing effect of misonidazole and hyperbaric oxygen. *Int. J. Radiat. Oncol. Biol. Phys.*, **12**, 1283.

ROCKWELL, S. & LOOMIS, R. (1980). Effects of sodium pentobarbital on the radiation response of EMT6 cells *in vitro* and EMT6 tumors *in vivo*. *Radiat. Res.*, **81**, 292.

ROFSTAD, E.K. (1981). Radiation response of cells of a human malignant melanoma xenograft. Effect of hypoxic cell radiosensitizers. *Radiat. Res.*, **87**, 670.

ROFSTAD, E.K. (1984). Growth and vascular structure of human melanoma xenografts. *Cell Tissue Kinet.*, **17**, 91.

ROFSTAD, E.K. (1985). Human tumour xenografts in radiotherapeutic research. *Radiother. Oncol.*, **3**, 35.

ROFSTAD, E.K. (1986*a*). Growth and radiosensitivity of malignant melanoma multicellular spheroids initiated directly from surgical specimens of tumours in man. *Br. J. Cancer*, **54**, 569.

ROFSTAD, E.K. (1986*b*). Radiation biology of malignant melanoma. *Acta Radiol. Oncol.*, **25**, 1.

ROFSTAD, E.K., DEMUTH, P. & SUTHERLAND, R.M. (1988*a*). $^{31}P$ NMR spectroscopy measurements of human ovarian carcinoma xenografts: Relationship to tumour volume, growth rate, necrotic fraction and differentiation status. *Radiother. Oncol.*, **12**, 315.

ROFSTAD, E.K., FENTON, B.M. & SUTHERLAND, R.M. (1988*b*). Intracapillary $HbO_2$ saturations in murine tumours and human tumour xenografts measured by cryospectrophotometry: Relationship to tumour volume, tumour pH and fraction of radiobiologically hypoxic cells. *Br. J. Cancer,* **57,** 494.

ROFSTAD, E.K. & SUTHERLAND, R.M. (1988). Radiation sensitivity of human ovarian carcinoma cell lines *in vitro*: Effects of growth factors and hormones, basement membrane, and intercellular contact. *Int. J. Radiat. Oncol. Biol. Phys.*, **15,** 921.

ROFSTAD, E.K., WAHL, A. & BRUSTAD, T. (1986*a*). Radiation response of multicellular spheroids initiated from five human melanoma xenograft lines. Relationship to the radioresponsiveness *in vivo. Br. J. Radiol.,* **59,** 1023.

ROFSTAD, E.K., WAHL, A., DAVIES, C. DE L. & BRUSTAD, T. (1986*b*). Growth characteristics of human melanoma multicellular spheroids in liquid-overlay culture: Comparisons with the parent tumour xenografts. *Cell Tissue Kinet.,* **19,** 205.

SUIT, H.D., SEDLACEK, R.S., SILVER, G. & DOSORETZ, D. (1985). Pentobarbital anesthesia and the response of tumor and normal tissue in the C3Hf/Sed mouse to radiation. *Radiat. Res.,* **104,** 47.

SUTHERLAND, R.M. & DURAND, R.E. (1973). Hypoxic cells in an *in vitro* tumour model. *Int. J. Radiat. Biol.,* **23,** 235.

SUTHERLAND, R.M. & DURAND, R.E. (1976). Radiation response of multicell spheroids – an *in vitro* tumour model. *Curr. Top. Radiat. Res. Q.*, **11,** 87.

SUTHERLAND, R.M. & FRANKO, A.J. (1980). On the nature of the radiobiologically hypoxic fraction in tumors. *Int. J. Radiat. Oncol. Biol. Phys.,* **6,** 117.

SUTHERLAND, R.M., SORDAT, B., BAMAT, J., GABBERT, H., BOURRAT, B. & MUELLER-KLIESER, W. (1986). Oxygenation and differentiation in multicellular spheroids of human colon carcinoma. *Cancer Res.,* **46,** 5320.

WEST, C.M.L., SANDHU, R.R. & STRATFORD, I.J. (1984). The radiation response of V79 and human tumour multicellular spheroids – cell survival and growth delay studies. *Br. J. Cancer,* **50,** 143.

WEST, C.M.L. & SUTHERLAND, R.M. (1987). The radiation response of a human colon adenocarcinoma grown in monolayer, as spheroids, and in nude mice. *Radiat. Res.,* **112,** 105.